# Design of Reinforcement in Nano- and Microcomposites

**DOI:** 10.3390/ma12091474

**Published:** 2019-05-07

**Authors:** Małgorzata Chwał, Aleksander Muc

**Affiliations:** Institute of Machine Design, Cracow University of Technology, 31-155 Cracow, Poland; olekmuc@mech.pk.edu.pl

**Keywords:** optimization, homogenization, microcomposites, nanocomposites, FEA, evolutionary algorithms, the shape of the reinforcement

## Abstract

The application of numerical homogenization and optimization in the design of micro- and nanocomposite reinforcement is presented. The influence of boundary conditions, form of a representative volume element, shape and distribution of reinforcement are distinguished as having the crucial influence on a design of the reinforcement. The paper also shows that, in the optimization problems, the distributions of any design variables can be expressed by *n*-dimensional curves. It applies not only to the tasks of optimizing the shape of the edge of the structure or its mid-surface but also dimensional optimization or topology/material optimization. It is shown that the design of reinforcement may be conducted in different ways and 2D approaches may be expanding to 3D cases.

## 1. Introduction

The group of non-homogeneous materials includes, e.g., composite materials, liquid mixtures, media with liquid and solid particles, as well as porous media. Reflecting deeper on the structure of a material, one can conclude that all materials are heterogeneous on a given scale. However, for some media, inhomogeneity can be observed only after moving to the atomic scale. Heterogeneity affects the physical processes occurring in the selected medium and causes difficulties in the description of such a medium. Due to the above, the idea of determining, if possible, a macroscopically equivalent medium has emerged. 

In place of a non-homogeneous medium in nano- or microscale, a homogeneous medium in macro-scale is introduced. The terms of the microscopic and macroscopic medium concern the distinction of the scale of heterogeneity from the scale of homogeneity. The method of theoretically moving from a heterogeneous scale to a homogeneous scale is called homogenization. Generally, the homogenization techniques deliver the properties of composite considering the properties of constitutes and their volume fractions. The homogeneous body with effective material properties is introduced instead of the heterogeneous body [1,2,3]. The two most straightforward and typical models of micromechanics are the parallel and series models yielding the upper and lower bounds of the composite properties. The parallel model introduced by Voigt [4] for the estimation of the average constants for polycrystals is based on a uniform strain and gives the upper-bounds (the known rule of mixture). The assumption of Reuss [5] also known as the series model is based on constant stress and gives the lower-bounds [2]. These approximations consider only the elastic properties of constitutes and their volume fractions. In the homogenization procedure, we can also distinguish direct and indirect approaches. In the direct homogenization, the volume averaging of stress, strain and energy is applied and the effective properties are calculated according to the definition of effective properties of the composite [6]. This approach is applied in numerical (finite element method—FEM) homogenization for calculation of local field quantities, whereas the geometry and properties of microstructure can be arbitrary. In the indirect homogenization, the Eshelby’s equivalent inclusion method is used [7]. The effective properties of the composite are computed based on volume fraction and geometry of the reinforcement as well as the properties of components. Hill [8] developed the self-consistent method for a single ellipsoidal inclusion embedded in an infinite medium. The author presented that the stiffness tensor components for composite lie somewhere in the interval between the Reuss and Voigt bonds, regardless of the geometry. Later, Christensen [9] presented the generalized self-consistent scheme. Composite constituents act together, and the material properties are the result of their interaction. Thus, Hashin and Shtrikman [10] proposed how to determine narrower bonds for the homogenized elastic properties for composite with isotropic constituents taking into account also the interaction between the elastic properties. They applied the principle of minimum potential energy and the idea of polarization. Halpin [11] presented Hill’s moduli in the more straightforward analytical form known as Halpin–Tsai equations and extended its application to a wide variety of reinforcement geometries [12]. Mori and Tanaka [13] proposed that the average strain in the interacting inclusions can be approximated by that of a single inclusion (fiber) embedded in an infinite matrix subjected to the uniform average matrix strain. This method was next reformulated by Benveniste [14]. In Mori–Tanaka theory, concentration matrices are given by the solution of a single inclusion in an infinite matrix. Taking into account a two-phase composite with an ellipsoidal inclusion, the Eshelby’s tensor is applied to the determination of the elastic field in a particle. Explicit expressions of the components of Eshelby’s tensor are presented in work by Mura [15]. Both Bensoussan et al. [16] and Suquet [17] discussed an asymptotic homogenization theory being a mathematical description of periodic composites. Aboudi [18] has presented a unified micromechanical theory using interacting periodic cells to predict the overall behavior of composites. The homogenization is also applied in the characterization of nanocomposites [19,20,21,22,23,24,25,26,27,28,29,30,31,32,33,34].

Using the homogenization theory, we can determine the effective mechanical properties of a representative volume element (RVE) made of fiber and matrix. The shape of the edge of the area occupied by the fiber has a significant effect on the homogenized mechanical properties for the elementary cell. Consequently, it also affects the stiffness of the laminate. Therefore, the design of the composite involves not only the tasks of optimizing the shape of the edge of the structure or its mid-surface but also dimensional optimization or topology/material optimization—see, e.g., references [35,36,37,38]. The functionally gradient material (FGM) distributes the material functions throughout the material body to achieve the maximum mechanical properties [39,40,41]. FGMs are mostly characterized as structures having different properties in the thickness direction; however, other propositions consider density grading in a width or length direction. Moreover, diagonal grading (in two directions) is also discussed.

The application of the optimization in homogenization problems has been introduced by Bendsoe and Kikuchi [42] and later discussed in papers, e.g., Refs. [43,44,45,46,47]. The homogenization method provided much valuable information used not only in the theoretical description but also in engineering practice. The use of homogenization methods helps in the design of micro- and nanocomposite materials with the desired properties.

The use of numerical homogenization and optimization in the modeling of micro- and nanocomposite reinforcement is presented here. From the mathematical and also numerical point of view, the description of microcomposites and nanocomposites is almost identical. The difference is only related to the real dimensions of the analyzed unit cells. The homogenization problem is solved based on the finite element method and compared with other approaches. It is shown that the design of reinforcement may be conducted in different ways and 2D approaches may be expanding to 3D cases. 

In this paper, the following issues influencing the reinforcement design in homogenization problem are discussed:

1. Boundary conditions of the representative volume element,
2. Form of the representative volume element,
3. Shape of the reinforcement,
4. Distribution of the reinforcement.

Points (3) and (4) are directly connected with the optimization of the 2D curves so the description of the analyzed problem (3) and (4) will be preceded by a short introduction on the 2D curve optimization. 

## 2. Preliminary Remarks

**Figure 1:**
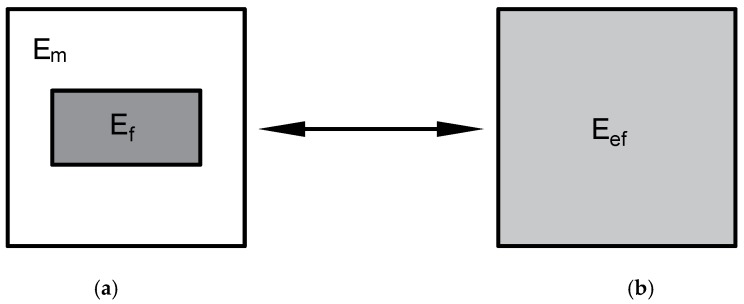
The homogenization problem: (**a**) heterogeneous and (**b**) equivalent homogenous body.

In homogenization, a heterogeneous body is replaced with a homogeneous one based on physical relationships between stresses and strains—Figure 1. Here, through a heterogeneous body, we understand the system in which there are two different phases, e.g., matrix and reinforcement. In contrast, a homogeneous system is understood as the system that characterizes effective material properties. 

The effective Hook’s law for a composite is defined as:

(1) $${\bar{\sigma }}_{ij}=\left[C^{*}\right]{\bar{\varepsilon }}_{ij},$$

where the overbar denotes the average over the unit cell and ${\bar{\sigma }}_{ij}$ and ${\bar{\varepsilon }}_{ij}$ is the average stress and strain tensor, respectively, $\left[C^{*}\right]$ is the effective elastic modulus, for which a total number of independent components is controlled by the prescribed symmetry, *i*, *j* = 1,2,3.

In the micromechanical analysis, the geometry of the arrangement of fibers in the matrix is an essential condition to develop a material model for the analysis. In the present study, it is assumed that the fibers and the matrix are only two phases in the composite, the fibers and matrix are perfectly bonded, fibers are aligned and have a uniform diameter along its length. Therefore, the transverse isotropy is assumed. Both constituents in the composite comply with Hooke’s law. 

The transversely isotropic material is characterized by a set of five equations having five effective independent stiffness moduli $C_{11}^{*}$, $C_{12}^{*}$, $C_{22}^{*}$, $C_{23}^{*}$, $C_{44}^{*},$ which can be expressed in terms of five independent engineering constants, such as the axial and transverse Young’s moduli $E_{A}^{*}$ and $E_{T}^{*}$, the axial and transverse Poisson’s ratios $\nu_{A}^{*}$ and $\nu_{T}^{*}$, and the axial shear modulus $G_{A}^{*}$—see Ref. [2].

In the numerical homogenization, the average stresses and strains are calculated using the volume averaging procedure:

(2) $${\bar{\sigma }}_{ij}=\frac{1}{V}\int \sigma_{ij}dV=\sum_{k=1}^{N}\sigma_{ij}^{k}V^{k},$$

(3) $${\bar{\varepsilon }}_{ij}=\frac{1}{V}\int \varepsilon_{ij}dV=\sum_{k=1}^{N}\varepsilon_{ij}^{k}V^{k},$$

where *N* is a total number of integration points in the unit cell, $\sigma_{ij}^{k}$, $\varepsilon_{ij}^{k}$ are stress and strain component, respectively, defined at integration points *k* and $V^{k}$ is the connected volume. 

## 3. Boundary Conditions

The three classical boundary conditions applied in the analysis of RVE include:

- Linear displacement boundary condition (Dirichlet condition),
- Constant traction boundary condition (Neumann condition),
- Periodic boundary condition.

Historically, the micromechanical models are the mechanical or engineering models. In the 1970s, a mathematical equivalent to micromechanical methods appeared, being the asymptotic homogenization theory. The fundamentals of asymptotic theory can be found, e.g., in Sanchez-Palencia [48], Benssousan et al. [16], and Suquet [17], among others. The asymptotic homogenization theory used periodic boundary conditions in the modeling of composites. Suquet [17], among others, showed that the “plane-remains-plane” boundary conditions are over-constrained conditions. It was shown that characteristic modes of deformation do not result in plane boundaries after deformation. Firstly, used “plane-remains-plane” boundary conditions on RVEs are only valid for the symmetric RVE loaded by normal traction, e.g., Xia et al. [49]. Homogenization theory, which implies the periodic boundary conditions, conducts to the more accurate results than the use of homogeneous displacement and homogeneous traction boundary conditions, e.g., Nguyen et al. [50]. Periodic boundary conditions are simplified to ordinary boundary conditions for the symmetric RVEs [6].

Considering the numerical homogenization, the effective strain and tangent operator associated with the strain tensor at any point in the macro scale are computed by the formulation of the boundary problem related to the microscale at that point [50]—see Equations (1)–(3). In the numerical homogenization (finite element method), the constitutive macroscopic assumption is not required, but, in this case, the issue of the appropriate use of periodic boundary conditions emerges, e.g., Sun and Vaidya [51]. The periodic boundary conditions are formulated as constrained equations of displacement differences for each pair of the opposite parallel boundary surfaces of the RVE. The periodic boundary conditions are the most efficient in terms of convergence rate from the three kinds of boundary condition for the growing size of the RVE [50].

In the present studies, we have focused on the numerical homogenization based on the finite element analysis of RVE in 2D and 3D problems. After solving the assumed numerical problem for specific displacement/force boundary conditions, the distribution of stresses and strains at each point of the inhomogeneous two-phase medium are obtained. The stress/strain values are variable, i.e., $\sigma_{ij}=\sigma_{ij}\left(x,y,z\right)$ and $\varepsilon_{ij}=\varepsilon_{ij}\left(x,y,z\right)$. The homogenization of such a body requires the assumption of a specific material model of an equivalent homogeneous body to determine the required number of equivalent material constants. There is a full analogy here with the methodology of experimental studies of anisotropic bodies. To demonstrate the finite element (FE) approach, we discuss a problem of transversaly isotropic composites—see also Chwał and Muc [31].

The displacements and tractions used at the boundaries of the RVE are generalized by [52]:

(4) $$u_{i}\left(S\right)=\varepsilon_{ij}x_{j},$$

(5) $$T_{i}\left(S\right)=\sigma_{ij}n_{j},$$

where *S* is the bounding surface, $x_{j}$ is the surface coordinate, and $n_{j}$ is the component of the normal vector to *S*. To obtain the material constants for assumed material model, the appropriate, e.g., displacement $u_{i}$ should be applied on the RVE that would produce uniform stresses $\sigma_{ij}$.

**Figure 2:**
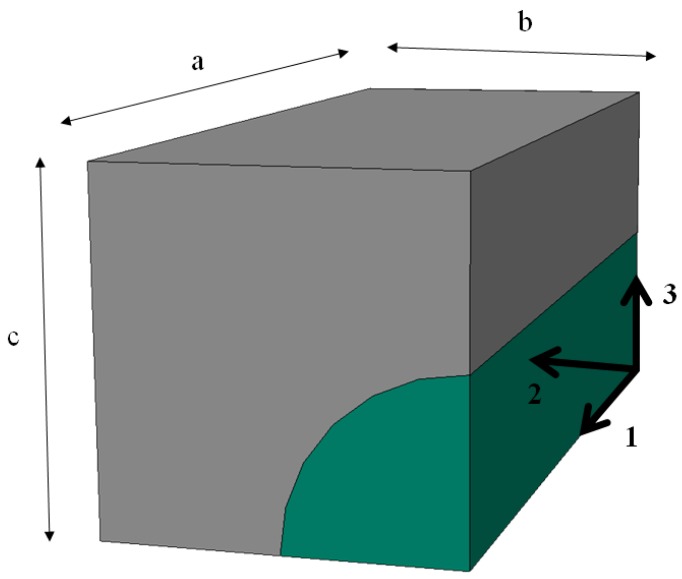
A quarter of representative volume element (RVE) having a square array of fibres with characteristic dimensions.

In numerical analyses, a series of FE simulations were carried out, in which RVEs were subjected to the various boundary displacements. The boundary conditions applied to the faces of the unit cells are presented in Table 1. The characteristic faces of the quarter of RVE are: *x*_1_ = 0, *x*_1_ = a, *x*_2_ = 0, *x*_2_ = b, *x*_3_ = 0 and *x*_3_ = c—see Figure 2. The *u*_1_, *u*_2_ and *u*_3_ denote the displacements along *x*_1_, *x*_2_ and *x*_3_ directions, respectively, and *e* is the assumed small displacement in the elastic regime. The effective material properties were computed using the appropriate boundary conditions (Table 1) and Equations (1)–(3).

Assuming an 2D orthotropic body, there are four independent material constants, i.e., axial and transverse Young’s moduli, in-plane Poisson ratio and in-plane shear modulus which are determined experimentally by three types of tests: (i) elongation in a direction parallel to the fiber, (ii) elongation perpendicular to the fiber; and (iii) shear in-plane. In numerical homogenization of such a body, it is necessary to build three independent numerical models that perform elongation in a direction parallel to the fiber, elongation in the direction perpendicular to the fiber and in-plane shear. We determine the average values of stresses and strains in both directions—Equations (2) and (3), and then, using the physical equation, we calculate the necessary material constants. 

## 4. Form of the Representative Volume Element

The distribution of reinforcement in micro- and nanocomposites is of high attention and has a significant influence on their mechanical behavior. In fibrous composite materials, the fiber distribution is mostly random and usually unknown. Thus, some idealizations of fiber distribution in the polymeric matrix are assumed in the analysis of composite materials.

**Figure 3:**
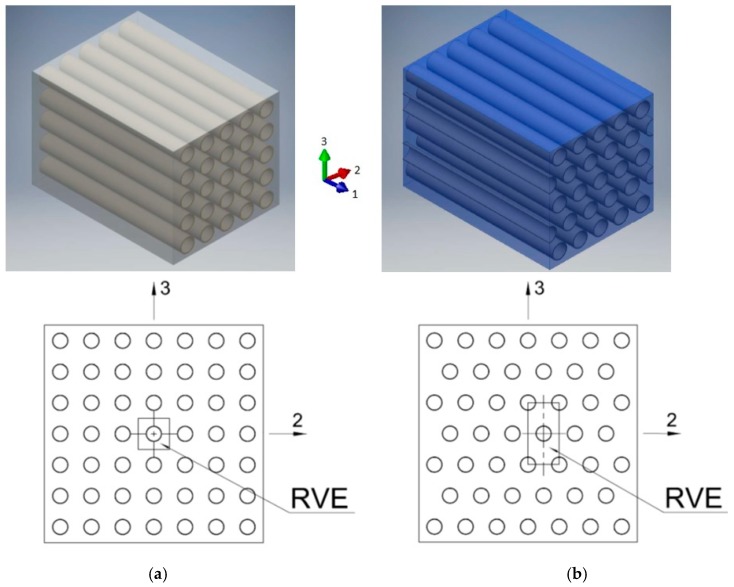
Schematic diagram of unidirectional fibres distribution in a matrix and selected representative volume elements: (**a**) square; (**b**) hexagonal array, respectively.

According to Hill [53], in the perfect distributions of fibers, it is seen that, due to symmetry and periodicity of these arrays, one representative array can be selected to analyze the lamina at the microscale. Thus, from the whole specimen, a representative subregion called representative volume element (RVE) can be selected—Figure 3. Additionally, this RVE as a volume of material statistically characterizes a homogeneous material. The above approach is used in an analysis of microcomposites and nanocomposites.

There are many ways to idealize the cross-section of a lamina [54,55]. The usually preferred arrangements of unidirectional fibers in the matrix are square and hexagonal arrays. Two popular idealizations are presented in Figure 3a,b. The nine independent constants are presented from the 3D constitutive equations considering unidirectional lamina being the orthotropic material in nature. Additionally, for a transverse isotropic body, there are five independent constants. Here, it is assumed that the isotropic plane (2–3) is perpendicular to the axial direction (1) of the fibrous reinforcement—Figure 3a.

Here, the attention is focused on the characterization of elastic properties of epoxy nanocomposites with a small amount of carbon nanotubes involving FE modeling contrasted with classical micromechanical approaches. The present calculations consider our earlier studies on nanostructures [23,32,56,57,58,59,60] and nanocomposites [20,24,25,31]. The topic of polymeric nanocomposites with carbon nanotubes (CNTs) is widely discussed. Mostly the volume fraction of CNTs in nanocomposites is low. At higher CNT fractions, the mechanical properties of the nanocomposite were observed to deteriorate due to the formation of CNT agglomerates, which act as stress concentrators. Since the purpose of the analysis is to evaluate the material constants for nanocomposites, the prepared model should be able to simulate the stress–strain behavior. For this work, it was assumed that the reinforcement is in the form of straight aligned carbon nanotubes uniformly distributed in the epoxy matrix—Figure 3. The behavior of the reinforcement and the matrix was assumed to be isotropic, homogenous and linearly elastic. The typical values of stiffness moduli for carbon nanotube and epoxy resin have been used and are listed in Table 2. In the literature, the effect of carbon nanotubes alignment and waviness on the effective properties of nanocomposite is also discussed, see, e.g., the work by Savvas et al. [34]. 

To compute the effective properties of CNT/epoxy nanocomposite, 3D quarter unit cells for square and hexagonal arrays were built and analyzed in the ABAQUS package. The typical dimensions for the quarter of RVE with a square array of CNTs are presented in Figure 3. The same characteristic notations have been used for RVE with a hexagonal array, namely: a—½ of the length, b—½ of the width, c—½ of the high of RVE. However, in the numerical analysis of nanocomposite, the nanotube has the circular cross-section with *R_i_*—the inner diameter of CNT, and *t*—the thickness of CNT—see Table 3. Other characteristic parameters of the conducted FEA are presented in Table 3.

**Figure 4:**
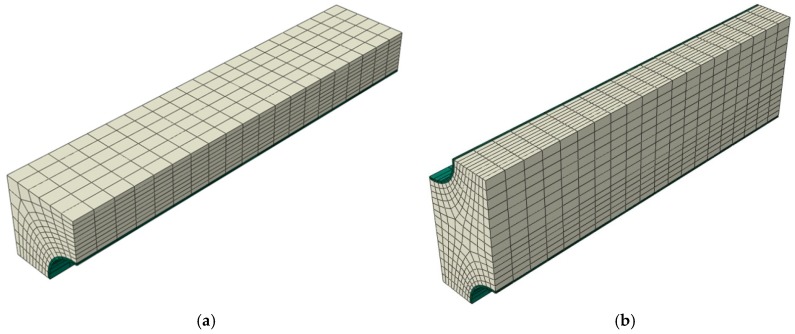
Finite element model of the 1/8 RVEs (**a**) square and (**b**) hexagonal arrays.

FE model of RVEs was built using 8-node linear hexahedrons with reduced integration—C3D8R solid finite elements available in the ABAQUS package. A double layer of elements on the CNT was used for both square and hexagonal RVE—Figure 4. The general structural analysis was conducted.

The results of the current calculation of the effective engineering constants of CNT/polymer nanocomposites are listed in Table 4.

The present results based on the numerical homogenization for the square and hexagonal arrays are contrasted with results from micromechanical models such as Vanin model [61] (see Equation (A1)). The analytical micromechanical computations were conducted based on the boundary conditions presented in Table 1.

For axial Young’s modulus $E_{A}^{*}$ and Poisson’s ratio $\nu_{A}^{*}$, FEA results are almost identical for the square and hexagonal arrays and are the same as from the rule of mixture applied in the Vanin model. Other elastic moduli, the transverse Young’s modulus $E_{T}^{*}$, the axial shear modulus $G_{A}^{*}$ and the transverse Poisson’s ratio $\nu_{T}^{*}$ represent the main challenge for the researchers. Here, the predicted values of $E_{T}^{*}$, from FE studies are higher than the micromechanical predictions. The current FE model leads also to the relatively high value of the axial shear modulus $G_{A}^{*}$ that is not predicted by micromechanical models. Lower discrepancy is observed for the transverse shear modulus $G_{T}^{*}$. The transverse Poisson’s ratio $\nu_{T}^{*}$ calculated numerically for the square and hexagonal arrays show higher values than computed according to micromechanical model.

## 5. Optimization Problems with the Use of 2D Curves

**Figure 5:**
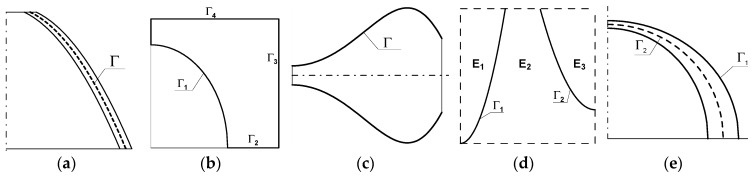
Description of 2D optimization problems with the use of 2D curves: (**a**) shape optimization—the shape of the middle surface is described by the curve Γ; (**b**) shape optimization—the curve describes boundaries of structures $\Gamma =\Gamma_{1}\cup \Gamma_{2}\cup \Gamma_{3}\cup \Gamma_{4}$; (**c**) dimensional optimization—structure thickness distribution is described by the curve Γ; (**d**) material optimization—a rectangle is made of three different composite materials (the curves $\Gamma_{1}$ and $\Gamma_{2}$ characterize the division of the area); (**e**) optimization of the shape of the mid-surface $\Gamma_{2}$ and dimensional optimization (the curve $\Gamma_{1}$ describes the distribution of the thickness).

In optimization problems, distributions of arbitrary design variables can be expressed through n-dimensional curves. This applies not only to the problems of optimizing the shape of the edge of the structure or its central surface but also dimensional optimization (e.g., thickness) or topology/material optimization (distributions: density, the volume fraction of strengthening in the material, the material with different Young’s modulus)—see Figure 5. In the latter case, the curves form the boundary between materials with different physical and chemical properties. In cases of simultaneous analysis of optimization, e.g., shape and dimensional, we introduce two types of curves: one describes the variations of the shape of the middle surface, and the other changes the thickness—see Figure 5e.

In this area, optimization of the shape of the external edges of structures (structural shape optimization) is still the most developed in both the 2D and 3D approaches—Figure 5b. Historically, the first work in this field concerned the optimization of the structure’s edge—see Ramakrishnan and Francavilla [62]. Currently, the main focus is on finite element (FE) formulations combined with the problems of automatic and even adaptive generation of FE mesh, analytical, semi-analytical or numerical sensitivity analysis (see, e.g., Barthelemy and Haftka [63]) and the development of the effective analytical or numerical methodology of the curve Γ generation.

By the curve, we understand a geometrical figure for which a parametric description can be entered. Generally, it is a set of p points that satisfy the condition p = p(t), where t ∈ [a, b]. The parametric description of the curve in the *n*-dimensional space requires the specification of *n* functions that describe the coordinates of the points of this curve. The method of description (representation) of the curve is directly related to the number of design variables included in the optimization process. Typically, three different variants are used:

- Set of nodes’ approximates curve Γ (see, e.g., Lee et al. [64]) created by discretization of finite elements, then the number of design variables is large and equal to the number of nodes on the edge Γ, and additionally increases in cases of description concentration of stresses; this method is used very rarely.
- Curve Γ is described by elementary analytic functions, in which case it is unambiguously defined by giving a finite fixed number (see Pedersen [65,66]), much smaller than in the previous case; however, there is always a problem with the introduction of the function and assessment, whether it is sufficient to solve a specific optimization problem; this method has unfortunately too little generality.
- Application of spline functions to the definition of curve Γ, where the spline functions are uniquely determined by specifying the coordinates of a finite number of key points P*_i_* (*i* = 0,1,2, ..., *I*); the number of basis points is equivalent to the number of design variables.

Currently, most of the optimization problems are related to the need for FE discretization of both the curve Γ and the entire structure to determine the value of the objective function. The accuracy of the solution of the problem depends on the accuracy of the approximation of the curve and the numerical model created for the specific task of optimization. 

In the further part of the work, we will discuss issues related to two-dimensional curves, i.e., *n* = 2. We do not intend to discuss here the fundamental aspects of optimization algorithms. They are presented in details in Refs. [67,68,69].

### 5.1. Representation of the Curve Γ through Elementary Functions

**Figure 6:**
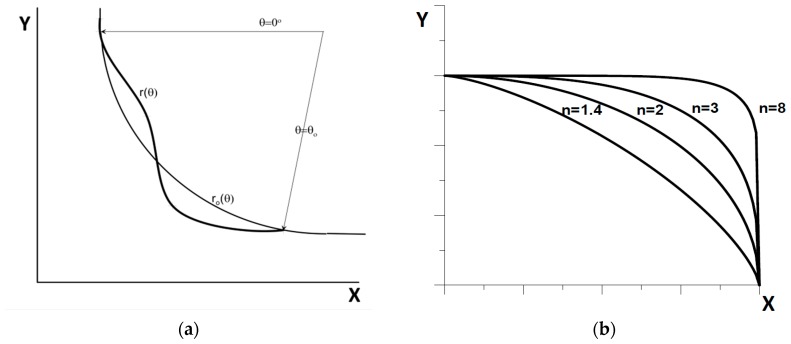
(**a**) parameterization of the curve Γ through eigenfunctions in regions of stress concentration; (**b**) superellipse shapes for different values of parameter n.

The simplest method of building the curve Γ is to express it through analytic functions and a set of parameters. Variations in parameters allow us to optimize the shape. The a priori unknown number of parameters necessary to describe the problem is a distinct disadvantage of such an approach. This methodology can be treated as a starting point for a thorough analysis using spline functions. Already in 1976, Kristiansen and Madsen [70] presented the method of representing the curve in the polar coordinate system in the form:

(6) $$r\left(\theta \right)=r_{0}\left(\theta \right)+\sum_{i=1}^{P}t_{i}f_{i}\left(\theta \right),\;\;\;\;\;\;\;0\leq \theta \leq \theta_{0},$$

where the function $r_{0}\left(\theta \right)$ defines the basic shape (most often it is an ellipse or a circle—see Figure 6a), and $f_{i}\left(\theta \right)$ are eigenfunctions that solve the beam vibration equation *f*^IV^ − *λf* = 0:

(7) $$f_{i}\left(\theta \right)=cosh\left(k_{i}\theta \right)-cos\left(k_{i}\theta \right)+a\left[sinh\left(k_{i}\theta \right)-sin\left(k_{i}\theta \right)\right],\;\;\;\;\;k^{4}=\lambda ,$$

where *a* is a constant resulting from the accepted conditions on the edges θ=0 and $\theta =\theta_{0}$, and the parameters $t_{i}$ (real numbers) form a set of design variables.

Pedersen et al. [65] extended the number of design variables in the problem of shape optimizing by introducing in Equation (6) unknown parameters of the curve $r_{0}\left(\theta \right)$ defining the basic shape. They proposed a description of this curve as a superellipse whose equation in the Cartesian system has the form:

(8) $${\left(\frac{x}{a}\right)}^{n}+{\left(\frac{y}{b}\right)}^{n}=1,$$

where *a*, *b*, *n* are additional design variables—see Figure 6b. The use of superellipse enables the description of various shapes of the reference curve Γ starting from the straight line (*n* = 1) and ending with the square (*n* → ∞). A wider variety of basic shapes is introduced in this way. The curves described by Equations (6) and (8) are used in conjunction with FE models and numerical sensitivity analysis for solving specific optimization tasks. 

### 5.2. Generation of Key Point Population

The number of key points and the way they are arranged in a two-dimensional coordinate system (equidistant or not) are directly dependent on the type of optimization problem. It is obvious that the increase in the number of key points increases the size of the task and is unfavourable. On the other hand, increasing the number of key points allows a better representation of the curve Γ. The authors’ experiences show that it would be expedient to introduce 5 to 10 basis points to describe one curve. The issue of choosing the location of key points depends primarily on the type of imposed equality or inequality constraints. However, the initialization of the task always starts from the equidistant position of the points. For all key points, we will additionally introduce inequality constraints in the form of:

(9) $$r_{min}\leq r_{i}\leq r_{max},\;\;\;\;\;\;i=0,1,2,\ldots ,I,$$

where *I* specifies the number of key points, and $r_{min}$ and $r_{max}$ are determined depending on the demand; these may be, for example, technological requirements. The values of real numbers $r_{i}$ are determined using a zero-one pseudo-random number generator. We treat the numbers $r_{i}$ as the length of the radii at the end of which the key point *P_i_* is located. In the adopted polar coordinate system, it is necessary to define the angular positions of the radii $r_{i}$. Below are some options for defining them depending on the imposed additional restrictions. It should be emphasized that, in this case, limitations are not boundary conditions imposed at the ends of curve Γ. They are taken into account directly in the methodology of its construction. 

#### 5.2.1. No Constraints—Equidistant Key Points

**Figure 7:**
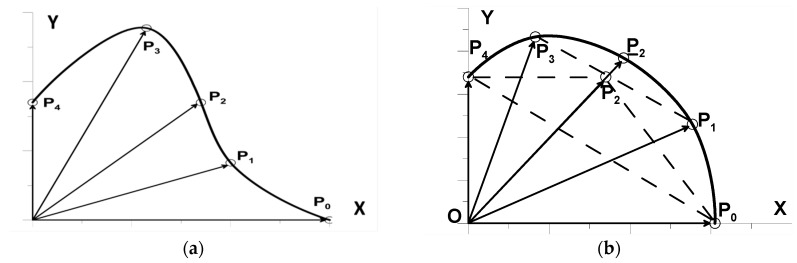
(**a**) generation of key points in the absence of constraints (five basis points); (**b**) construction of convex curves (five basis points).

We assume that it is possible to change the angular position of the key points in the range [0°,90°]. We divide this interval into equally-spaced sections. It is assumed that the first of the generated key points always lie on the OX axis. The vectors start at the origin of the coordinate system. An example of such a generation is shown in Figure 7a.

In the tasks of the curve generation, we are primarily interested in the shape of the geometric figure Γ geometry, and not its location in a particular FE model. In specific numerical cases, it is always possible to translate and rotate the curve to place one (or two) base vectors in the required position.

#### 5.2.2. Generation of Convex Curves

In many optimization problems, the convexity of the generated curve Γ is required. The idea of creating convex curves directly uses the convex polygons’ construction algorithm. We randomly generate *I* + 1 equidistant (in the sense of angular distance) base vectors $r_{i}$. Next, we create a new sequence of base vectors, starting with the vector with the smallest (largest) length, depending on whether the vector with the smallest (largest) length was generated first in the original sequence. Such a method of selecting the first base vector provides the ability to construct curves for which the base radius on the OX axis may be smaller or larger than the radii on the OY axis. This operation was carried out for the vectors presented in Figure 7a. The new sequence of base vectors (P_0_,...,P_4_) is shown in Figure 7b. Next, we check if the vectors form a convex polygon, investigating whether the vector associated with the P_i_ point is above or below the line joining the points (P_i−1_, P_i+1_). If it is below the straight line, then we generate a new length so that the end of the vector is beyond the straight line. We start checking the condition of the convexity from the inside, i.e., by examining the position of the vector P_2_ relative to the two extreme ones, i.e., P_0_ and P_4_. We examine the positions of further points analogously, i.e., dividing subsequent intervals into halves. Of course, the most comfortable is to take an odd number of key points. After completing these operations, repeat the verification of the protuberances relative to the neighbors. In the case shown in Figure 7b, the point P_2_ is above the line joining points P_0_ and P_4_, but not with respect to the neighboring P_1_ and P_3_. Thus, it is necessary to generate a new key point (by generating a new length of the vector) marked in Figure 7b as P_2_ Currently, the set of key points forms a convex polygon, and we can construct a convex curve Γ. In computer graphics, the non-uniform rational basis spline (NURBS) mathematical model is commonly used for generating and representing curves and surfaces. In NURBS, control points are entered and the edition of the curve is realized based on the element’s control points for Bézier curves or based on spline modeling.

#### 5.2.3. Generation of a convex Curve with Constraints in the Form of a Constant Area Bounded by a Curve Γ

Now, let us discuss the construction of the convex curve Γ with the constraint in the form of the constancy of the *A* field bounded by this curve and with pre-set boundary conditions in the form of the prescribed values of the angle of tangents at the edges of the curve, i.e., at the key points $P_{0}$ and $P_{I}$. The boundary conditions will be fulfilled as if automatically by determining suitably the positions of the control points necessary for the construction of the curve segments by cubic spline functions. It is a task independent of the generation of key points since such a generation is treated only and exclusively as a problem in the field of geometry. We start the construction of the curve from the generation of the pseudo-random position of the $P_{0}$ point on the OX axis, taking into account the Equation (9). Depending on the values of prescribed angles, we distinguish two cases:

(a) one of the angles on the edge of the curve is 0° and the other 90°,
(b) one of the angles on the edge of the curve is not equal to 0° or 90°.

In the first case (a), the length of the vector $r_{I}$ (the position of the $P_{I}$ point on the OY axis) is determined initially assuming that the searched curve is a quarter of an ellipse with a given area *A*, i.e.,:

(10) $$r_{I}=\frac{4A}{\pi r_{0}}.$$

The length of this vector should also satisfy Equation (9). The angular positions of the remaining key points are uniquely defined at this stage of the construction, and later they will not be changed anymore. Numerical analysis has shown that the equal angular distances of the base vector positions are not suitable for convex curve construction in the case of a small number of key points. For this reason, we suggest calculating angular positions of base vectors using the information about the arc length of an ellipse with semi-axes ($r_{0}$, $r_{I}$). It is assumed that the ellipse segments, obtained after the division of the angle 90° into the *I* parts, have equal lengths of arcs. It can be approximately assumed (with sufficient numerical accuracy) that the length of each segment of the ellipse is equal to:

(11) $$l_{i}=\frac{\pi \left[1.5\left(r_{0}+r_{I}\right)-\sqrt{r_{0}r_{I}}\right]}{4I},\;\;\;\;\;i=1,2,\ldots ,I.$$

In the second case (b), the length of the vector $r_{I}$ (the position of the *P_I_* point on the OY axis) is determined by assuming initially that the searched curve is described as the so-called Ferguson curve, which in parametric form is expressed by the formula:

(12) $$\left[\begin{array}{c} x\left(t\right)\\ y\left(t\right) \end{array}\right]=\left[\begin{array}{c} r_{0}\\ 0 \end{array}\right]\left(1-3t^{2}+2t^{3}\right)+\left[\begin{array}{c} 0\\ r_{I} \end{array}\right]\left(3t^{2}-2t^{3}\right)+\left[\begin{array}{c} x^{\prime }\left(t=0\right)\\ y^{\prime }\left(t=0\right) \end{array}\right]\left(t-2t^{2}+t^{3}\right)+\left[\begin{array}{c} x^{\prime }\left(t=1\right)\\ y^{\prime }\left(t=1\right) \end{array}\right]\left(-t^{2}+t^{3}\right),\;\;\;\;\;t\in \left[0,1\right],$$

where the prime denotes the derivative concerning the parameter *t*. As previously, it is assumed that the area under the Ferguson curve is equal to *A*. The angular positions of the key points are determined from a similar condition as for case (a). Next, for the geometrical construction, we generate pseudo-randomly the remaining lengths of base vectors, demanding that two conditions be satisfied:

1. Convexity of a polygon stretched on basis vectors,
2. Boundary conditions at the ends of the curve.

**Figure 8:**
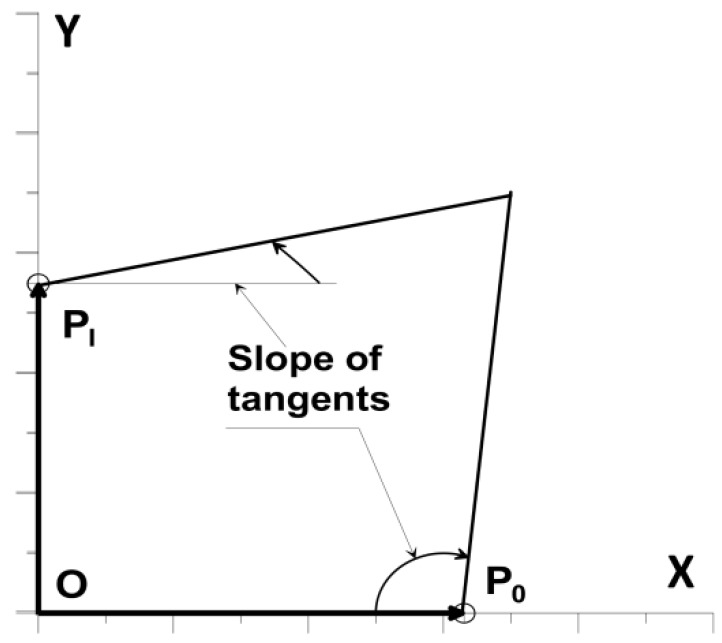
The area of acceptable positions of base vectors inside a quadrangle.

The numerical implementation of the first condition is analogous to that described in the previous chapter. The second condition imposes only geometric constraints on the area of possible positions of base vectors, as shown in Figure 8.

After selecting the positions of base vectors, it is necessary to check the condition of the constant area bounded by a polygon. The relationship for calculating the triangle area OP_i−1_P_i_ is used here:

(13) $$A_{\Delta P_{i-1}P_{i}}=\frac{1}{2}r_{i-1}r_{i}sin\left(∠P_{i-1}OP_{i}\right).$$

If the sum of the fields of individual triangles is not equal to *A*, then we reduce or increase the value of the radius r_I_ and repeat the operations of selecting the positions of the vectors starting from the correction of the angular positions of the base vectors. The construction of the curve that meets the condition of the convexity and constancy of the area is a set of geometrical observations based on the idea of rejecting solutions (positions) that do not meet the imposed conditions (unsuccessful trials). This method is simple for numerical algorithms and, in our opinion, has large generalisations in the sense of being easily adaptable to a range of similar problems.

## 6. Shape of the Reinforcement

### 6.1. 2D Homogenization Problem

**Figure 9:**
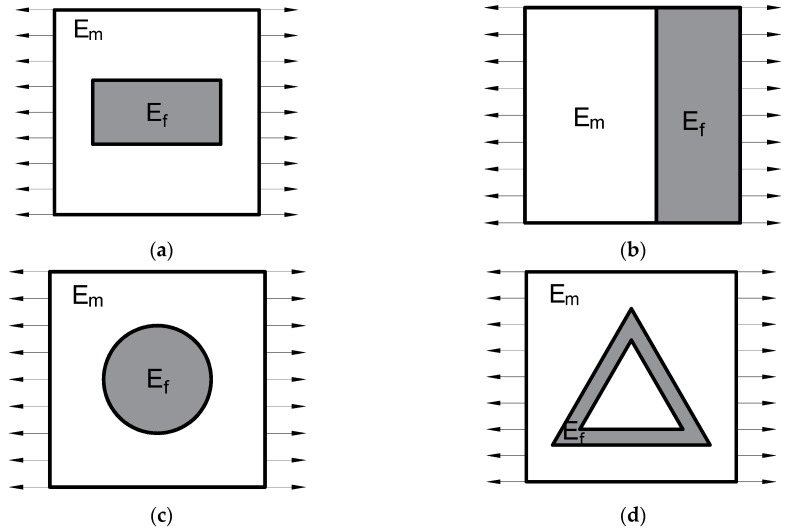
2D RVE: (**a**) a structure with a uniform distribution of short fibre; (**b**) a bilayered structure; (**c**) a structure with a circular reinforcement; and (**d**) a structure with a triangular shape of reinforcement.

The homogenization of 2D microcomposites has been presented for several examples of RVE, i.e., a structure with short fibre (Figure 9a), a bilayered structure (Figure 9b), a structure with a circular reinforcement (Figure 9c), and a structure with a triangular shape of reinforcement (Figure 9d). The results of numerical homogenization and values calculated from micromechanical models have been presented and discussed. The problem of the inclusion shape and its influence on the effective properties of heterogeneous media is especially emphasized in the literature, e.g., in the paper by Savvas et al. [71].

The numerical homogenization procedure was applied to analyze 2D composites with the uniform distribution of short fibres (Figure 9a), with the bilayered structure (Figure 9b), a structure with a circular reinforcement (Figure 9c), and with the triangular shape of reinforcement (Figure 9d). The assumed material data are listed in Table 5, and volume fraction of reinforcement for the short fibres, circular reinforcement and the bilayered structure was $v_{f}=32\%,$ whereas, for the triangular shape of the reinforcement, $v_{f}=10\%$. The analytical solution for the problem of 2D composites with the uniform distribution of short fibres is presented, e.g., in the paper by Fuji and Zako [72]. Here, a short fibre having ratio *d*/*l* = 0.5 was analyzed (Figure 9a). The uni-axial loading in the elastic regime was applied for all 2D RVEs—Figure 9 and plane strain elements (CPE4 in ABAQUS package) were used. 

In the 2D analysis of microcomposites, the effective Young’s modulus $E^{*}$ in the loading direction as the ratio of average stresses ${\bar{\sigma }}_{ij}$ to average strains ${\bar{\varepsilon }}_{ij}$ (Equations (2) and (3)) was computed numerically. The $E^{*}$ evaluated numerically was compared with rule of the mixture (ROM), Halpin-Tsai (H-T) model for short fibers, Cox model [73] and micromechanical model presented by Strzelecki [74] (see Equations (A2)–(A5)). The results are listed in Table 6. For all analyzed cases, the effective Young’s modulus according to the rule of mixture is higher than the numerical values. The numerical homogenization results are close to the Halpin-Tsai model for short fibres, whereas the Cox model gives lower values of $E^{*}$. The FEM values of $E^{*}$ for the bilayered stucture are very close to the micromechanical models. Similarly, as in cases (a) and (c)—Figure 9, the numerical results of RVE with the triangular shape of the reinforcement are much lower than the values from the rule of mixture. The applied micromechanical models give higher results, but the difference is not so high. However, it should be noted that the shape of the reinforcement is very complicated compared to those adopted in micromechanical models. This evokes other distributions of stresses and strains in the representative cell and thus causes differences in the relation to simple micromechanical models. Practically, such a complicated shape excludes the possibility of using these models due to difficulties in determining the diameter of the reinforcement. Here, the reporting results in Table 6 assumed *d*/*l* = 0.075 in analytical computations. 

The values of the equivalent Young’s modulus obtained from the numerical analysis for the 2D problems presented above were significantly different from the results calculated based on the rule of mixture. The application of the simple rule of mixture overestimates the value of the effective Young’s modulus (see Table 6). The use of micromechanical models is appropriate in the case of simple shape reinforcement, i.e., an easy determination of the reinforcement diameter and length. In the case of a complicated shape of the reinforcement (see Figure 9d), phenomenological models should not be used because the determination of *d*/*l* ratio is problematic.

### 6.2. Isoperimetric Problem—Verification of the Accuracy of Numerical Solutions

If we impose equality conditions limiting the sought functions, then the optimization of the shape (looking for the extremum of a function dependent on the function defining the shape of the structure’s edge) with imposed constrained on the shape function (in the implicit and functional form) are the classic, isoperational variational problems. The solutions of these tasks are sought in the class of differentiable functions with a given metric and satisfying the imposed equality constraints—see Tatarkiewicz [75]. One of the most well-known issues in this field is the following problem:

*Find a closed plane curve of a given perimeter L (refers to the length of the boundary connecting two points A and B) which encloses the maximal Area.*

The solution is a circle (the class of curves is limited to smooth curves) with an unknown location of the center of the circle and radius. These quantities are determined from the boundary conditions.

**Figure 10:**
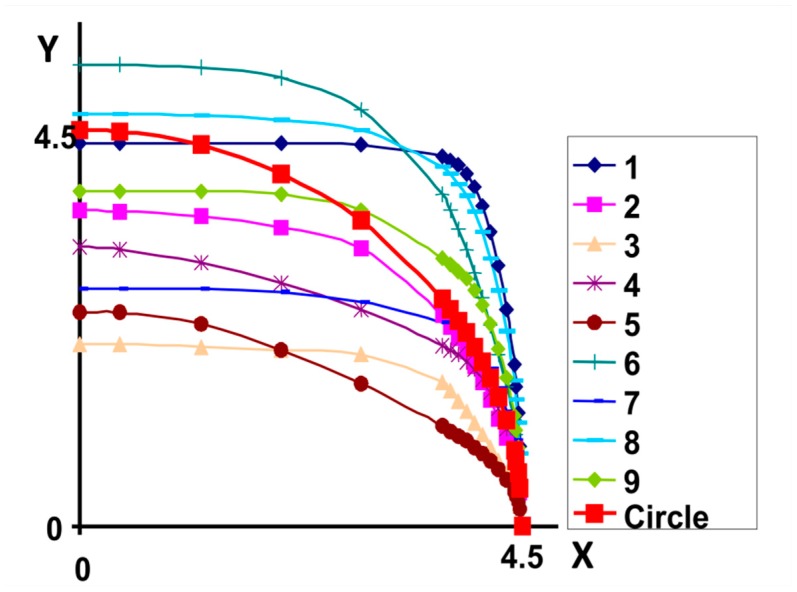
Generation of nine elements of the initial population with the solution of the Equation (14).

The above problem is very convenient to check to what extent the application of Bezier’s spline functions is sufficient to approximate continuous convex functions. However, to obtain a specific numerical solution, it is necessary to specify constants. We assume that the center of the circle is located at the beginning of the Cartesian coordinate system and the arc length of the circle *L* connecting the points A and B is πr, where r is half the length of the segment AB. The solution to the problem is, therefore, a semi-circle with an area of πr^2^/2. Due to the symmetry of the problem concerning the *y*-axis, we only look for half of the curve. Finally, we formulate the optimization problem in the following way:

*Find*

(14) $$Max\left[Area-\eta \left(\tilde{L}-L\right)\right],$$

where *Area* means the field under the convex curve Γ with the arc length $\tilde{L}$, and *η* the penalty parameter. This amount was assumed in the calculations to be equal to 250. It was also assumed that the curves sought are always convex. Examples of a generation of nine different populations are shown in Figure 10. The area of the surface under each of the generalized curves is equal to the area of the circle.

**Figure 11:**
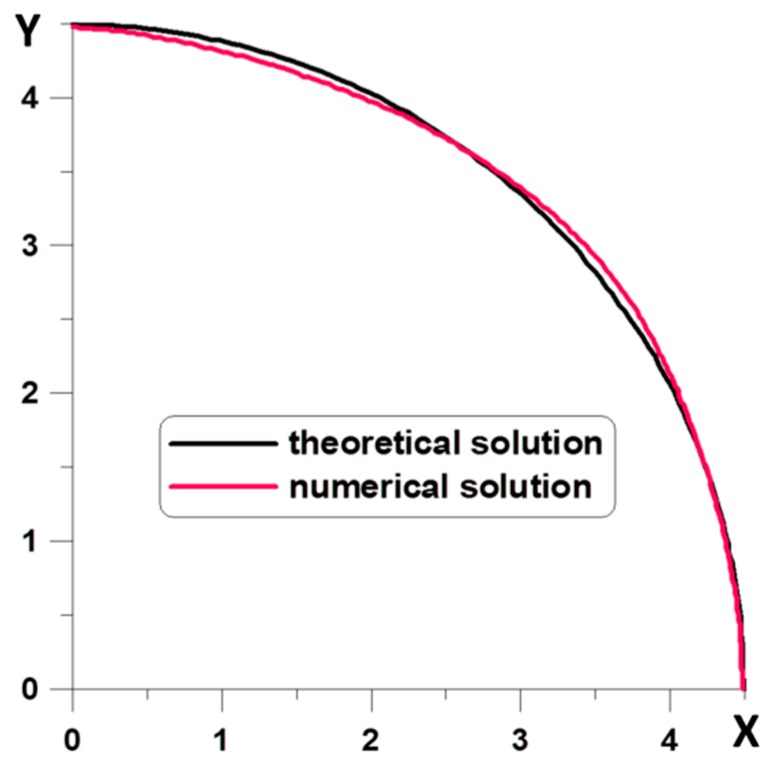
Optimal curves—GA and MEA (black color) and obtained using the optimization procedure (red color).

The results of calculations for a population of 50 individuals are shown in Figure 11. In the calculations, it has been assumed that functions must always pass through a point with coordinates (4.5,0.0). Furthermore, the possible coordinate variations in the vertical direction were limited, i.e., 0.0 ≤ *y* ≤ 1.2 × 4.5 = 5.4.

As can be seen, in Figure 11 and Table 7, there is a good agreement of theoretical and numerical solutions, both in the case of using genetic algorithms (GA) as well as a modified evolutionary strategy (MEA).

**Figure 12:**
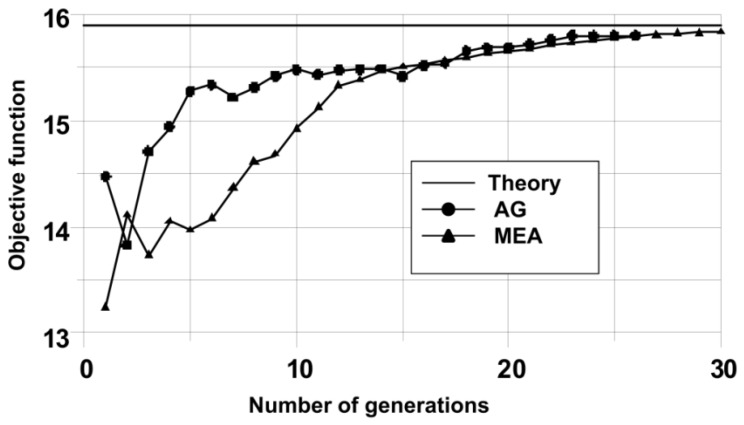
Convergences of the optimization.

The number of generations necessary to obtain optimal solutions is small—see Figure 11. It is mainly due to the imposition of a constraint in the form of a demand to fulfil the condition that curves Γ are to be always convex. In this way, generated curves that do not meet this condition are not taken into account in the results presented in Figure 12.

### 6.3. Shape of Fibre Bundles

Unidirectional composites are not reinforced with a single fiber but a bundle of fibers embedded in the matrix. The shape of the cross section of the fiber bundle determined as the edge of the area occupied by the fiber $\Gamma =\partial \Omega_{f}$ can be arbitrary, i.e., it can form a circle, an ellipse, etc. Using the numerical homogenization (see Section 2 and [31]), we can determine the mechanical properties of an elementary cell made of fiber and matrix. The area occupied by the cell is denoted as $\Omega =\Omega_{f}\cup \Omega_{m}$. The shape of the edge of the area occupied by the fiber has a significant effect on the homogenized mechanical properties for the elementary cell. Thus, it also affects the stiffness of the laminate. The purpose of the optimization is to determine the edge shape $\Gamma =\partial \Omega_{f}$, *to minimize the function of the total internal energy of the RVE* in the Lagrange form, i.e.,:

(15) $$Min\Pi_{int}^{RVE}$$

with a constraint that the area enclosed by the curve Γ is constant, i.e.,:

(16) $$\int_{\Gamma =\partial \Omega_{f}}g_{\Gamma }\left(x,y\right)dxdy=A_{f}=const,$$

where $g_{\Gamma }$ defines a function describing the curve. Equation (15) can be written in the following explicit form:

(17) $$\Pi_{int}^{RVE}=\frac{1}{2}\left[C_{ijkl}^{m}+\left(C_{ijkl}^{f}-C_{ijkl}^{m}\right)\rangle A_{kl\alpha \beta }\langle \right]\varepsilon_{\alpha \beta }\varepsilon_{ij}.$$

We approximate the curve Γ using the spline functions by entering five key points. The condition in the form of Equation (16) will be written in the dimensionless form during the generation of key points. In the numerical analysis, we consider only 1/4 of the elementary cell. Therefore, additional restrictions on the derivative value at the ends of the curve were imposed.

**Figure 13:**
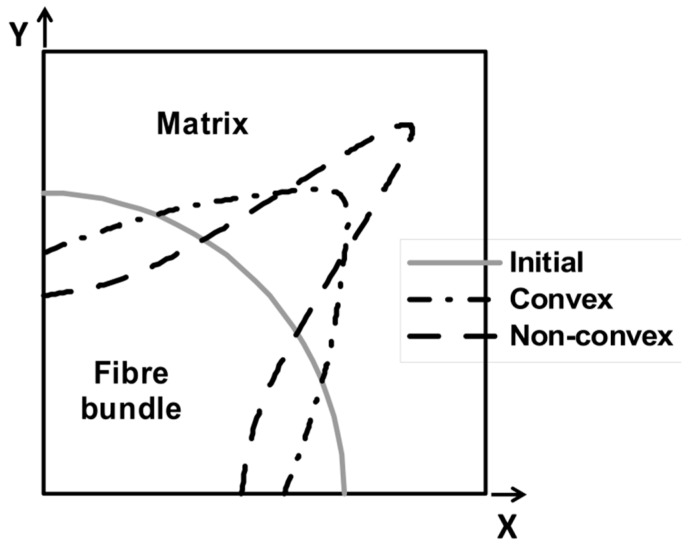
Planar representative volume element—initial (circular) and optimal shapes of fibre bundles.

The elemental cell was modeled using triangular FE type NKTP 1 (plane stress state—NISA package). On the edge of the elementary cell, external exertion was applied in the form of a unit displacement acting along the *x*-axis. A circle was assumed as the original shape of the fiber bundle. The results of solving Equations (15) and (16) are shown in Figure 13. In the numerical computations, the algorithm of the modified evolutionary strategy (MEA) was used. The shape of the fiber bundle depends on the conditions limiting the form of the curve Γ understood in the sense of its convexity or not.

The presented numerical solution draws new possibilities to optimize the mechanical properties of the material by forming a bundle of reinforcement fibers. It is possible from a technological point of view. However, attention should be paid to the issue of the interfacial surface, which was not included in the considerations. The shape of the fiber bundle may have some influence on the initialisation process of the micro failure at the fiber/matrix boundary and may be responsible for the formation of macrocracks and destruction of the material.

## 7. Distribution of the Reinforcement

The functionally gradient materials (FGM) distribute the material functions throughout the material body to achieve the maximum mechanical properties. FGMs are characterized as structures having different properties in the thickness *z*-direction in the form proposed by Qian et al. [39]:

(18) $$p\left(z\right)=\left(p_{t}-p_{b}\right)V\left(z\right)+p_{b},$$

where *p* denotes a generic material property like modulus, *p_t_* and *p_b_* denote the corresponding properties of the top and bottom faces of the plate, respectively, and *V*(*z*) is a function describing the distribution of reinforcement density in the thickness direction. For FGM, the function *V*(*z*) is defined as:

(19) $$V\left(z\right)={\left(\frac{z}{t}+\frac{1}{2}\right)}^{n}\;\;\;z\in \left[-\frac{t}{2},\frac{t}{2}\right]$$

Other definitions of the *V*(*z*) can be found, e.g., in reference [40]. Other propositions consider the use of Equations (18) and (19) for degradation in a different direction than thickness. First density grading in the width direction is as follows:

(20) $$\rho \left(x\right)=\rho_{l}-\frac{x\left(\rho_{l}-\rho_{t}\right)}{w}$$

where *ρ_l_* and *ρ_t_* are the mass densities of the leading edge and trailing edge, respectively; *w* denotes the width of the panel. Second density grading in the length direction is as follows:

(21) $$\rho \left(x\right)=\rho_{r}-\frac{x\left(\rho_{r}-\rho_{t}\right)}{L}$$

where *ρ_r_* and *ρ_t_* are the mass densities of the root edge and tip edge, respectively; *L* denotes the length of the panel. Next definition of density diagonal grading across the *xy* plane (in two directions) is as follows:

(22) $$\rho \left(x,y\right)=\frac{L-x}{L}\left(\frac{w-y}{w}\rho_{u}+\frac{y}{w}\rho_{l}\right)+\frac{x}{L}\left(\frac{w-y}{w}\rho_{l}+\frac{y}{w}\rho_{u}\right)$$

where *ρ_u_* and *ρ_l_* are the mass densities of upper and lower bounds, respectively. A model for characterizing the mechanical properties of functionally graded materials (FGMs) with the regular polygonal cross-section is also developed by introducing the power-law rule—see Ref. [41]. However, modelling of their mechanical behaviour still leads to many problems, particularly in the description of 1D or 2D structures, such as beams, plates or shells. The broader discussion of those problems and solutions can be found in Muc et al. [76,77,78,79,80].

**Figure 14:**
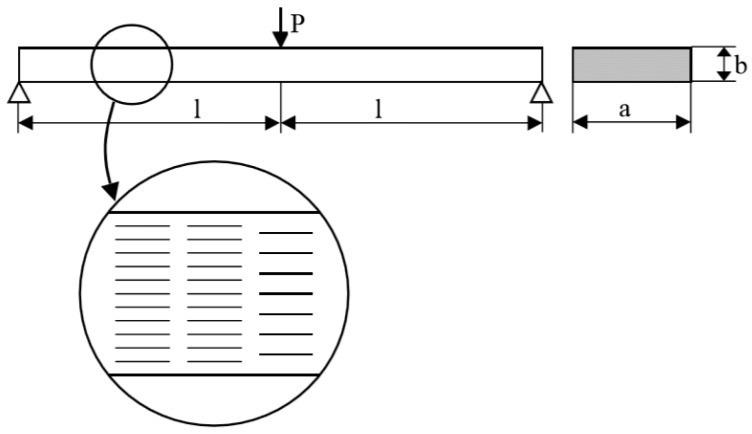
Bending of a simply supported beam having a variable fibre volume fraction distribution along the length.

Let us consider a composite beam reinforced by short fibres, loaded at the center (Figure 14—three-point bending test) and having a variable density of fibres $\rho_{f}\left(x\right)$ along the length. The present methodology is similar to the FGM description. In the current problem, the density grading in the length direction is presented.

**Figure 15:**
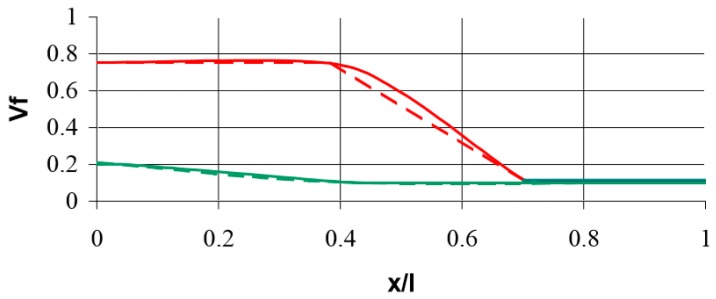
Comparison of fibre volume fraction distribution along the beam length: analytical (a dotted line) Banichuk et al. [81] and numerical (a continuous line) results.

The objective of the optimisation is as follows: *to find the minimal mass of the beam satisfying the equality constraint imposed on the displacement parameter and inequality (upper and lower bounds) constraint—on the fibre volume density fraction* V_f_—the strict formulation is given by Banichuk et al. [81]. The optimisation problem deals with the topology optimisation since we are looking for the optimal material distribution. The results are presented in Figure 15 and show a good correlation between numerical and analytical studies. Upper values (red lines) in Figure 15 were calculated for the lower value of the assumed beam deflection. 

## 8. Conclusions

The present paper discussed the application of numerical homogenization and optimization in the studies of micro- and nanocomposites. The issue of boundary conditions, reinforcement shape and distribution, and form of representative volume element is presented taking into account also the topic of 2D curves optimization.

The conducted analyses and comparisons are summarized as follows:

- The method of description (representation) of the 2D curve related to the number of design variables included in the optimization process was presented.
- There was a good agreement of theoretical and numerical solutions, both in the case of using genetic algorithms (GA) as well as a modified evolutionary strategy (MEA) for the shape optimization of the single fibre.
- In shape optimization of fibre bundles, a circle was assumed as the original shape. In the numerical computations, the algorithm of modified evolutionary strategy (MEA) was used. The shape of the fibre bundle depends on the conditions limiting the form of the curve Γ understood in the sense of its convexity or not.
- The composite beam reinforced by short fibres, loaded at the centre and having a variable density of fibres along the length, was considered, being an example of FGM. The optimization problem deals with topology optimization since we were looking for the optimal material distribution. The results showed a good correlation between numerical and analytical studies.
- Some examples of numerical homogenization of various 2D and 3D RVE have been studied to present the influence of boundary conditions, form of RVE, shape and distribution of the reinforcement on the effective material properties.
- The comparison of numerical homogenization and micromechanical models showed that micromechanical models are appropriate in the case of the simple shapes of reinforcement.
- However, for complicated shapes of the reinforcement, the application of the numerical homogenization is crucial because the determination of characteristic relations such as d/l ratio is problematic.

## Figures and Tables

**Table 1:** Boundary displacements of the quarter of representative volume element.

Loading	Constants	Boundary Displacements					
		*x*_1_ Direction		*x*_2_ Direction		*x*_3_ Direction	
		*x*_1_ = 0	*x*_1_ = a	*x*_2_ = 0	*x*_2_ = b	*x*_3_ = 0	*x*_3_ = c
Axial normal	$E_{A}^{*}$ and $\nu_{A}^{*}$	*u*_1_ = 0	*u*_1_ = *e*	*u*_2_ = 0		*u*_3_ = 0	
Transverse normal	$E_{T}^{*}$ and $\nu_{T}^{*}$	*u*_1_ = 0		*u*_2_ = 0	*u*_2_ = *e*	*u*_3_ = 0	
Axial shear	$G_{A}^{*}$	*u*_2_ = 0 *u*_3_ = 0	*u*_2_ = *e* *u*_3_ = 0	*u*_1_ = 0 *u*_3_ = 0	*u*_1_ = 0 *u*_3_ = 0	*u*_3_ = 0	*u*_3_ = 0

**Table 2:** Material properties of carbon nanotube and epoxy resin used in finite element analysis.

Material Constant	Carbon Nanotube	Matrix
Young’s modulus (GPa)	1000	3.2
Poisson’s ratio	0.3	0.3

**Table 3:** Parameters used in FE modeling of nanocomposite RVEs.

**Parameter**	**Value**	
Outer diameter of CNT (nm)	10	
Thickness of CNT (nm)	0.3	
Length of CNT (nm)	100	
Volume fraction of CNTs	2.75%	
**Parameter**	**Square Array**	**Hexagonal Array**
Dimensions of RVE: 2a × 2b × 2c (nm)	100 × 20 × 20	100 × 20 × 40
Total number of FE for the whole RVE	30,880	36,160
Element type	C3D8R—8-node linear hexahedrons with reduced integration	

**Table 4:** Comparison of the effective engineering constants of CNT/polymer nanocomposites.

Model	$E_{A}^{*}$ (GPa)	$E_{T}^{*}$ (GPa)	$\nu_{A}^{*}$	$\nu_{T}^{*}$	$G_{A}^{*}$ (GPa)	$G_{T}^{*}$ (GPa)
FEM–square array	30.62	3.90	0.3	0.6	4.29	1.22
FEM–hexagonal array	30.60	4.15	0.3	0.56	4.16	1.32
Vanin model	30.61	3.62	0.3	0.41	1.3	1.28

**Table 5:** Elastic properties of microcomposite constituents.

Material Constant	Reinforcement	Matrix
Young’s modulus (GPa)	70.4	3.2
Poisson’s ratio	0.22	0.35

**Table 6:** Effective Young’s modulus of microcomposites (material data in Table 5).

2D RVEs	Effective Young’s Modulus (GPa)		
	FEM	Micromechanical Models	Rule of Mixture (Equation (A2))
bilayered structure	9.301	9.119—(Equation (A3))	24.704
align short fibre	8.26	3.798—Cox (Equation (A5)) 8.777—H-T (Equation (A4))	24.704
triangular shape of the reinforcement	4.588	6.987—Cox (Equation (A5)) 7.280—H-T (Equation (A4))	9.92
circular inclusion	5.635	-	24.704

**Table 7:** Comparison of the numerical results.

Notation	Theoretical Solution	The Type of Algorithm	
		Genetic Algorithm	Modified Evolutionary Strategy
The length of the arc—$\tilde{L}$	7.06858	7.06113 Error: 0.11%	7.06344 Error: 0.073%
Area—*Area*	15.90431	15.7987 Error: 0.66%	15.83521 Error: 0.43%

## Appendix A

Vanin model

(A1) $$E_{11}^{*}\approx V_{t}E_{t}+V_{m}E_{m}+\frac{8{\left(\nu_{t}+\nu_{m}\right)}^{2}V_{t}V_{m}G_{m}}{1+V_{m}+V_{t}\delta_{m}+V_{m}\left(\delta_{t}-1\right)\frac{G_{m}}{G_{t}}},\;G_{12}^{*}=G_{13}^{*}\approx G_{m}\frac{1+V_{t}+V_{m}\frac{G_{m}}{G_{t}}}{V_{m}+\left(1+V_{t}\right)\frac{G_{m}}{G_{t}}},G_{23}^{*}\approx G_{m}\frac{1+V_{t}+V_{m}\frac{G_{m}}{G_{t}}}{V_{m}+\left(1+V_{t}\right)\frac{G_{m}}{G_{t}}},\;\nu_{21}^{*}=\nu_{31}^{*}\approx \nu_{m}-\frac{V_{t}\left(\delta_{m}+1\right)\left(V_{t}-V_{m}\right)}{1+V_{m}+V_{t}\delta_{m}+V_{m}\left(\delta_{t}-1\right)\frac{G_{m}}{G_{t}}},\;\frac{1}{E_{22}^{*}}=\frac{1}{E_{33}^{*}}\sim \frac{\nu_{21}^{*2}}{E_{11}^{*}}+\frac{1}{8G_{m}}\left[\frac{2V_{m}\left(\delta_{m}-1\right)+\left(\delta_{t}-1\right)\left(\delta_{m}+V_{t}-V_{m}\right)\frac{G_{m}}{G_{t}}}{1+V_{m}+V_{t}\delta_{m}+V_{m}\left(\delta_{t}-1\right)\frac{G_{m}}{G_{t}}}+\frac{2G_{m}}{G_{23}^{*}}\right],\;\frac{\nu_{23}^{*}}{E_{22}^{*}}=-\frac{\nu_{21}^{*2}}{E_{11}^{*}}+\frac{1}{8G_{m}}\left[\frac{2G_{m}}{G_{23}^{*}}-\frac{2V_{m}\left(\delta_{m}-1\right)+\left(\delta_{t}-1\right)\left(\delta_{m}+V_{t}-V_{m}\right)\frac{G_{m}}{G_{t}}}{1+V_{m}+V_{t}\delta_{m}+V_{m}\left(\delta_{t}-1\right)\frac{G_{m}}{G_{t}}}\right],\;\delta_{i}=3-4\nu_{i}$$

where i=t or m denotes nanotube and matrix, respectively; $V_{t}$, $V_{m}$—volume fractions, $E_{t}$, $E_{m}$—Young’s moduli, $G_{t}$, $G_{m}$—Kirchhoff’s moduli, $\nu_{t}$, $\nu_{m}$—Poisson’s ratios.

Rule of mixture

(A2) $$E^{*}=v_{f}E_{f}+(1-v_{f})E_{m},$$

Strzelecki model:

(A3) $$E^{*}=\frac{E_{m}E_{f}}{v_{f}E_{m}+\left(1-v_{f}\right)E_{f}},$$

Halpin–Tsai (H–T) model for short fibres:

(A4) $$E^{*}=\frac{1+2\frac{l}{d}\eta_{L}v_{f}}{1-\eta_{L}v_{f}}E_{m},\;\eta_{L}=\frac{\frac{E_{f}}{E_{m}}-1}{\frac{E_{f}}{E_{m}}+2\frac{l}{d}},$$

Cox model:

(A5) $$E^{*}=\left(1-\frac{2tanh\left(\frac{\beta l}{2}\right)}{\beta l}\right)v_{f}E_{f}+\left(1-v_{f}\right)E_{m},\;\beta =\sqrt{\frac{4H}{\pi E_{f}d^{2}}},\;H=\frac{2\pi G_{m}}{ln\left(\frac{1}{\sqrt{v_{f}}}\right)}.$$
